# Psychotherapy mediated by remote communication technologies: a meta-analytic review

**DOI:** 10.1186/1471-244X-8-60

**Published:** 2008-07-22

**Authors:** Penny E Bee, Peter Bower, Karina Lovell, Simon Gilbody, David Richards, Linda Gask, Pamela Roach

**Affiliations:** 1 School of Nursing, Midwifery and Social Work, University of Manchester, Oxford Road, Manchester, M13 9PL, UK; 2 National Primary Care Research And Development Centre, University of Manchester, Oxford Road, Manchester, M13 9PL, UK; 3 Department of Health Sciences, University of York, University Road, Heslington, York, YO10 5DD, UK

## Abstract

**Background:**

Access to psychotherapy is limited by psychopathology (e.g. agoraphobia), physical disability, occupational or social constraints and/or residency in under-served areas. For these populations, interventions delivered via remote communication technologies (e.g. telephone, internet) may be more appropriate. However, there are concerns that such delivery may influence the therapeutic relationship and thus reduce therapy effectiveness. This review aimed to determine the clinical effectiveness of remotely communicated, therapist-delivered psychotherapy.

**Methods:**

Systematic review (including electronic database searching and correspondence with authors) of randomised trials of individual remote psychotherapy. Electronic databases searched included MEDLINE (1966–2006), PsycInfo (1967–2006), EMBASE (1980–2006) and CINAHL databases (1982–2006). The Cochrane Central Register of Controlled Trials (CENTRAL) and the Cochrane Collaboration Depression, Anxiety and Neurosis Controlled Trials Register (CCDAN-CTR). All searches were conducted to include studies with a publication date to July 2006.

**Results:**

Thirteen studies were identified, ten assessing psychotherapy by telephone, two by internet and one by videoconference. Pooled effect sizes for remote therapy versus control conditions were 0.44 for depression (95%CI 0.29 to 0.59, 7 comparisons, n = 726) and 1.15 for anxiety-related disorders (95%CI 0.81 to 1.49, 3 comparisons, n = 168). There were few comparisons of remote versus face-to-face psychotherapy.

**Conclusion:**

Remote therapy has the potential to overcome some of the barriers to conventional psychological therapy services. Telephone-based interventions are a particularly popular research focus and as a means of therapeutic communication may confer specific advantages in terms of their widespread availability and ease of operation. However, the available evidence is limited in quantity and quality. More rigorous trials are required to confirm these preliminary estimates of effectiveness. Future research priorities should include overcoming the methodological shortcomings of published work by conducting large-scale trials that incorporate both clinical outcome and more process-orientated measures.

## Background

Psychological disorders account for over 15% of the total burden of disease within established economies, a significant proportion of which manifests in depressive and anxiety-related disorders [1]. For these disorders, effective treatment options often include non-pharmacological as well as pharmacological interventions. Consensus guidelines recommend the use of cognitive-behavioral and interpersonal therapies for major depression [2-4] and cognitive behavioral therapy for panic disorder [5]. Research has also highlighted the potential value of these approaches in the treatment of dysthymia, phobia and generalized anxiety disorder [6].

Despite evidence of its efficacy however, most adults diagnosed with depressive or anxiety-related disorders do not receive psychotherapeutic care [6,7]. Insufficient numbers of mental health professionals impede access to effective interventions, with people living in remote or under-served areas often having to travel long distances to obtain face-to-face services [8]. Other recognized barriers include time and economic constraints, caring responsibilities, psychological or physical impairment and concerns regarding the potential stigma of attending outpatient appointments [9-11].

Remote communication technologies such as the telephone, internet or videophone have the potential to mitigate many of these inequalities [12,13]. The telephone in particular is a widely available telecommunication technology [14] that is being used increasingly as a mechanism for support and treatment delivery [15,16]. The establishment of 1–900 counseling services alone illustrates a long but ongoing commitment to its use within psychotherapy service provision. More recently, the emergence of computer-aided technology alongside growth in the popularity of the Internet have increased multifold the opportunities for real time, long-distance consultations [17].

Conventional wisdom still insists however, that for most purposes, psychological therapies should be delivered face-to-face. The central premise to this argument is that the effectiveness of such interventions depends upon on the development of a high quality therapeutic alliance between therapist and client [18]. Within the context of this relationship, visual as well as auditory information is reflected in behaviours such as eye contact, physical expression, posture and voice [19]. The use of the telephone or internet will invariably eliminate many of these cues whilst the use of teleconferencing will limit physical presence and touch. Whether or not an effective alliance can be delivered in the absence of interpersonal contact thus remains a topic of some debate [20-22].

Evidence to support the efficacy of non face-to-face psychotherapy service models is accumulating. Recent studies of collaborative care provision, in which telephone support is often provided as an adjunct to other interventions, have documented positive outcomes [23-27]. Reviews and meta-analyses of computer-delivered CBT packages [28,29] and self-help treatments [30-33] also provide promising results. These interventions however are often considered as stand-alone technologies requiring little or no contact between therapist and client. One recent meta-analysis of internet-based cognitive behavioral therapy for depression and anxiety [34] has suggested that the efficacy of these psychological interventions may ultimately be higher where therapist support is available.

The ability to deliver more intensive psychological therapy via remote communication media has the potential to confer multiple benefits for patients by combining real time, scheduled contact with increased accessibility. As yet however, the efficacy of delivering psychotherapy via such means has received comparatively little attention [19]. This article reports the findings of a systematic review conducted to determine the clinical effectiveness of remote psychotherapy.

## Methods

### Design

This research was conducted using systematic review techniques [35] and meta-analysis to assess the clinical effectiveness of psychotherapy delivered via remote communication.

### Inclusion criteria

#### Study design

Studies eligible for inclusion were published randomised controlled trials (RCTs). Due to a lack of financial resources for translation facilities, the review was restricted to English language publications. There were no specific methodological quality criteria for inclusion in the review. Instead data were extracted from all studies on key methodological issues (see 'Methods of the review' below).

#### Interventions

Interventions eligible for inclusion included any treatment incorporating a psychological intervention mediated by remote communication. Psychological interventions were defined as treatments with an explicit psychological orientation [36]. 'Mediated by remote communication' was defined as a treatment where all or the majority (i.e. with the exception of an initial or final contact meeting) of the psychological intervention was delivered by a therapist to a patient on a scheduled and repeated one-to-one basis through an appropriate technology (rather than face-to-face). Group and marital therapies or any other intervention involving interaction between a therapist and more than one client simultaneously were excluded from the review. Trials examining emergency crisis interventions (e.g. telephone helplines) or the efficacy of medication management programs in the absence of psychotherapy were excluded. Minimal interventions in which the majority of therapy was delivered i) outside of client-therapist interaction time (e.g. self-help therapy) or ii) via technology with little or no therapist contact (e.g. computerized CBT) were also excluded. Studies measuring changes in mental health symptoms as a by-product of counseling for physical illness were excluded.

#### Populations

Populations eligible for inclusion included any group seeking treatment for a mood disorder or functional (non-organic) mental health problem recognized by ICD-10 [37] or DSM criteria [38]. Studies involving therapy for substance misuse or addictions were excluded. Trials examining the efficacy of remote therapy for healthy populations *at risk *of mental health difficulties were also excluded.

### Search strategy

Electronic literature searches were performed using the Ovid electronic database on the MEDLINE (1966–2006), PsycInfo (1967–2006), EMBASE (1980–2006) and CINAHL databases (1982–2006). The Cochrane Central Register of Controlled Trials (CENTRAL) and the Cochrane Collaboration Depression, Anxiety and Neurosis Controlled Trials Register (CCDAN-CTR) were also searched. All searches were conducted to July 2006. Subject headings were used to identify all papers indexed as containing material relevant to mental/psychiatric health, psychological therapies/interventions and communication technologies. These headings were augmented by text words that included the full and abbreviated names of specific mental disorders, types of psychological therapy and modes of communication. Full details of the search terms used are appended.

Authors of published and ongoing studies were contacted for further studies and information on the progress of ongoing work: 50% replied with information. Reference lists from identified papers and from reviews in the area were searched by hand.

### Methods of the review

Eligibility judgements and data extraction were done independently by two reviewers. No formal measure of the reliability of data extraction was calculated but disagreements were resolved by discussion with other members of the project team.

A standardised data extraction recorded information on study context, population, interventions, outcomes and methodological quality. Methodological quality was assessed using standard criteria originally developed by the Cochrane Collaboration for Depression, Anxiety and Neurosis (CCDAN). The CCDAN quality rating scale [39] scores each study according to 23 elements of design and conduct including randomisation methods, sample size, follow-up period, power and appropriateness of analysis. Each criterion is scored from 0–2, giving a maximum score of 46.

The CCDAN criteria, like other validated scales of RCT quality [40], include a score pertaining to the adequacy of blinding procedures. Since it is not feasible to blind patients to an active intervention, this criterion was not relevant to the present review. A second criterion pertaining to the recording of pharmacological side-effects was similarly omitted from the review. Thus overall quality scores for the CCDAN criteria were in this study only able to reach a maximum of 42.

### Methods of analysis

All analyses were conducted using Comprehensive Meta Analysis [41]. Continuous measures were translated into a standardised effect size, Cohen's *d *[42]. Dichotomous variables were translated into standardised effect sizes by computing the log odds ratio, and then conversion to *d *using procedures in the meta analytic package. Initial meta-analyses used a fixed effect model [43] to provide an overall pooled measure of effect. Between-study heterogeneity was assessed using the I^2 ^statistic [44], which describes the percentage of total variation across studies that is due to heterogeneity rather than chance. The I^2 ^statistic has several advantages over other measures of heterogeneity, including greater statistical power to detect clinical heterogeneity when fewer studies are available. As a guide, I^2 ^values of 25% may be considered 'low', 50% 'moderate' and 75%, 'high'. Where heterogeneity was 'high' (i.e. I^2 ^values of 75% or above), a random effects model was employed.

For the purposes of analysis, three main comparison groups were created: i) remote communication therapy versus a control group (e.g. usual care, waiting list); ii) remote communication therapy versus conventional face-to-face therapy, and iii) different types of remotely communicated therapy. Where studies compared two different types of remote therapy with a control, both comparisons were entered into the meta-analysis separately, but control group sample sizes were halved to ensure that they were not double counted [45].

Studies examining depression underwent a separate analysis to those focussing on anxiety or anxiety-related disorders (e.g. anxiety, agoraphobia with panic, OCD). Studies assessed outcome over a range of time points. For the purposes of analysis, outcome assessments were categorised into two time periods, representing outcomes in the short (0–6 months), and longer term (7 months and over). Where studies reported assessments at multiple time points, data with the longest duration were used. Effect sizes were only calculated for the primary outcome measure (where specified), or the measure deemed most relevant to the mental health disorder under study (judged by KL and LG). When studies reported more than one relevant measure (e.g. BDI and HRSD), these were combined by taking an arithmetic mean [46].

## Results

Thirteen studies were included in the review. Of 40 studies identified but subsequently excluded, the most common reasons for rejection were i) a lack of a mental health primary outcome measure (n = 11); ii) the use of a minimal intervention (e.g. guided self help) or an intervention with no clear psychological orientation (n = 10); iii) the use of a technology-based therapy requiring no direct therapist involvement (n = 18) or iv) group therapy (n = 1). One further study was identified as ongoing and this may be of relevance for later versions of the review. Summaries of the included studies are given in table 1, with corresponding methodological details in table 2. A list of excluded studies is available from the authors.

**Table 1:** Interventions in the review

**Study**	**Target population**	**Study groups**	**Description of intervention in each group**
Hunkeler (2000)	Depressed primary care patients	Usual care plus telephone support & peer care	'Good care' incorporating regular GP visits, continued antidepressant prescribing and any other referral thought usual by GP. Augmented by telephone-delivered medication adherence support, side-effect discussions and behavioural activation plans (mean of 10.1 × 5.6 min sessions over 16 wks) plus one or more telephone or face-to-face (6/62 participants) peer support contacts.
		Usual care plus telephone support	As above, minus peer support
		Usual care	As above minus telephone & peer support.

Lange (2001)	Psychology students with trauma experience	Internet-mediated writing therapy	30 web-pages of psychoeducation followed by 10 × 45-min writing sessions over 5 wks (2/wk), therapist feedback (appro× 450 words) provided on 7 occasions across 3 treatment phases (self-confrontation, cognitive re-appraisal, sharing & farewell ritual).
		Waiting list	30 web pages of psychoeducation only

Lange (2003)	Individuals with mild-relatively severe trauma symptoms	Internet-mediated writing therapy	30 web-pages of psychoeducation followed by 10 × 45-min writing sessions over 5 wks (2/wk), therapist feedback (approx 450 words) provided on 7 occasions across 3 treatment phases (self-confrontation, cognitive re-appraisal, sharing & farewell ritual).
		Delayed treatment	As above, but only received once the intervention group had completed treatment.

Lovell (2006)	Secondary care outpatients with OCD	Face-to-face CBT	10 × 1-hr sessions using exposure & response prevention. Sessions incorporated the establishment of fear hierarchies, use of family co-therapist, weekly exposure targets (to be practised between sessions for at least 1-hr/dy), homework reviews and collaborative problem solving.
		Telephone CBT	8 weekly telephone calls of up to 30-mins in length with treatment content identical to above. Homework sheets posted to participants. Initial 1-hr face-to-face session covering the same material as the face-to-face arm plus 1 × 1-hr final session face-to-face

Lynch (1997)	Primary care patients with minor depression	Telephone counselling	6 × 20-min sessions based on problem-solving for depression; homework comprising of 5 steps of treatment including a demonstration of the connection between depressed mood and problems, expressing problems in a form that facilitates solutions, evaluating and modifying these solutions.
		Comparison group	No further details provided

Lynch (2004)	Primary care patients with minor depression	Telephone problem solving	Nezu's problem solving therapy adapted for telephone use and administered over a 6-wk period
		Telephone stress management	Treatment designed to serve as an attention control with topics including the identification of sources of stress, the importance of diet & exercise, ways of coping with stress
		Usual care	Usual treatment deemed appropriate by primary care physician.

McName e (1989)	Housebound agoraphobics with panic disorder	Telephone self exposure	Exposure goals set via 10 × 12-min telephone contacts with therapists. Subjects posted a self-help manual that encouraged use of coping strategies and family co-therapists.
		Telephone relaxation therapy	Subjects posted standard taped instructions of Jacobsen's relaxation and instructed to listen for at least 1-hr/dy. Therapy augmented by 10 × 12-min telephone consultations.

Miller (2002)	Women with history of recurrent/chronic depression	Telephone interpersonal psychotherapy (IPT-T)	12 × 1-hr scheduled weekly sessions.
		Usual care	No treatment beyond usual care

Mohr (2000)	Depressed MS patients	Telephone CBT	8 × 50-min sessions plus a workbook with assignments. Treatment delivered alongside access to usual care.
		Usual care	Any treatment given in the course of usual clinician care.

Mohr (2005)	Depressed primary care patients with MS	Telephone CBT (T-CBT)	Weekly 50-min sessions completed over 16 wks.
		Telephone supportive emotion focussed therapy (T-SEFT)	Weekly 50-min sessions completed over 16 wks

Nelson (2003)	Depressed children aged 8–14 yrs	Videoconferenc e CBT	8 sessions (1 × 90-min plus 7 × 60-min).
		Face-to-face CBT	8 sessions (1 × 90-min plus 7 × 60-min).

Simon (2004)	Depressed primary care patients	Telephone psychotherapy	8 × 30–40 min CBT plus 1 mail contact and 3 × 10–15 min telephone sessions focussed on medication management, caseload tracking and structured assessment.
		Telephone care management	As above minus telephone CBT. Patients given CBT self-management booklet but no further support provided.
		Usual care	No further details given

Swinson (1995)	Rural primary care patients suffering from panic disorder with agoraphobia	Telephone behaviour therapy	Mailed psychometric package and educational workbook serving as an introduction to behavior therapy concepts (e.g. hierarchy construction, exposure exercises, record keeping); 8 × 1-hr scheduled therapy sessions completed over approx. 10 wks. Therapy included exposure principles & exercises, long term goals, hierarchy construction, coping strategies, diary keeping, homework planning & reviewing.
		Waiting list	Initial psychometric package followed 10 wks later by an additional psychometric package and a workbook serving as an introduction to behavior therapy concepts (e.g. hierarchy construction, exposure exercises, record keeping).

**Table 2:** Characteristics of the included studies

**Study**	**Country**	**Target population**	**Recruitment**	**Sample ** **size**	**Outcomes**	**Follow-up**	**Follow-up ** **rate**	**CCDAN ** **score**
Hunkele r (2000)	US	Depressed primary care patients	GP referral	302	HAMD, BDI, SF-12	Baseline, 6 w, 6 m	90% at 6 w, 85% at 6 m	25

Lange (2001)	Netherlands	Psychology students with trauma experience	From student pool in return for course credits	30	IES, SCL-90, POMS	Baseline, 5 w, 11 w	83% at 5 w, 27% at 11 w	18

Lange (2003)	Netherlands	Individuals with mild-relatively severe trauma symptoms	Website contact	184	IES, SCL-90	Baseline, 5 w, 11 w	79% at 5 w, 31% at 11 w	21

Lovell (2006)	UK	Secondary care outpatients with OCD	Screening clinics	72	YBOCs, BDI	Pre-baseline, baseline, 1 m, 3 m, 6 m	90% at 6 m	36

Lynch (1997)	US	Primary care patients with minor depression	Screening	29	BDI, HAMD, DHP, PSI	Baseline, 7 w	55% at 7 w	20

Lynch (2004)	US	Primary care patients with minor depression	Screening	54	BDI, HAMD, DHP	Baseline, 6 w	57% at 6 w	17

McNam ee (1989)	UK	Housebound agoraphobics with panic disorder	Telephone screening	23	BDI, FQ, PT, GP, SA, GI	Baseline, 2 w, 4 w, 6 w, 8 w, 10 w, 12 w, 20 w, 32 w	78% at 6 w, 61% at 32 w	22

Miller (2002)	US	Women with history of recurrent/chronic depression	Ongoing longitudinal study	30	HRSD, GAS SAS-SR	Baseline, 12 w	80% at 12 w	22

Mohr (2000)	US	Depressed MS patients	Telephone screening	32	POMS	Baseline, 8 w	72% at 8 w	22

Mohr (2005)	US	Depressed primary care patients with MS	MS case registers & MS society newsletters	127	BDI, HDRS, PANAS	Baseline, 8 wk, 16 w, 3 m, 6 m, 9 m, 12 m	91% at 16 w	31

Nelson (2003)	US	Depressed children aged 8–14 yrs	Not clear	38	K-SADS-P, CDI	Baseline, 8 w	74% at 8 w	13

Simon (2004)	US	Depressed primary care patients	Computer records of patients starting new antidepressant treatment	600	SCL, PHQ	Baseline, 6 w, 3 m, 6 m	89% at 6 m	38

Swinson (1995)	Canada	Rural primary care patients suffering from panic disorder with agoraphobia	GP/family physician referral	46	FQ, STAI-T, BDI, ASI, SCL-90	Baseline, 10 w, 3 m	91% at 10 w, 76% at 3 m	20

Key: POMS-Profile of Mood States; BDI-Beck Depression Inventory; HAMD/HRSD-Hamilton Rating Scale for Depression; DHP-Duke Health Profile; PSI-Problem Solving Inventory; SCL-90-Symptom Checklist 90; STAI-T-State-Trait Anxiety Inventory (Trait version); ASI-Anxiety Sensitivity Index; PHQ-Patient Health Questionnaire; SCL-Hopkins Symptom Checklist Depression Scale; IES-Impact of Events Scale; GAS-Global Assessment Score; SAS-SR Social Adjustment Scale; K-SADS-P-Schedule for Affective Disorders & Schizophrenia for School Age Children-Present Episode; CDI-Children's Depression Inventory; PT-Phobic Targets; GP-Global Phobia; SA Social Adjustment; GI-Global Impression; FQ-Fear Questionnaire; YBOCs-Yale Brown Obsessive Compulsive Disorder Scale; PANAS-Positive & Negative Affect Scale; SF-12-Mental & Physical Composite Scales

### Scope of the included studies

Nine studies reported 11 comparisons of technology-mediated therapy versus a control, three studies focussing on anxiety-related disorders [47-49] and six on depression [50-55]. Four of these studies used usual care as the comparative arm and four a waiting list or no treatment control. The remaining study did not provide a clear description of its control group. Two studies reported comparisons of technology-mediated therapy with conventional face-to-face interventions, one in an anxiety-related disorders (OCD) [56] and one in depression [57]. Three studies reported comparisons between different types of therapy mediated by the same technology, one in anxiety [58] and three in depression [54,55,59].

Ten studies assessed the efficacy of psychological therapy mediated by telephone, two by internet and one by videoconference (Table 1). Participants were recruited via primary care screening or GP referral (five studies), secondary care outpatient screening (one study), by public advertisement (two studies), through case registers of Multiple Sclerosis patients (two studies), via longitudinal research (one study) and by access to a pool of psychology students (one study). One study focusing on depressed children did not provide a clear description of its recruitment context. Patients included those with PTSD, OCD, agoraphobia with panic disorder and depression (Table 2). The number of participants randomized within each trial ranged from 23 to 600 (mean 121), with losses at follow-up of between 9% and 73%. Length of follow-up ranged from 6 weeks to 1 year. No data on costs were reported in any of the studies.

### Quality of the included studies

All studies were RCTs, although in two studies methods of allocation were only quasi-randomised with the possibility of bias [50,58]. Only two out the 13 studies reported details of allocation concealment [55,56]. Less than half of the included studies (n = 5) defined a main outcome *a priori *[50,51,54-56] and only five reported conducting a power analysis [49,54-57]. Although all studies conducted largely or wholly appropriate analyses of their data this failed to include an intention to treat analysis in eight instances [47-51,54,57,58]. Seven studies controlled for between-group differences at baseline [47,48,51,53,55,56,59] and all but two studies presented sufficient data for re-analysis [50,57]. Only two studies involved more than 100 participants per trial arm [51,55], the vast majority (n = 9) recruiting less than 50.

The mean methodological quality score for the included studies was 23 (SD = 7.33). The highest scores were 38 [55], 36 [56] and 31 [59].

### Quantitative results

Pooled effect sizes for each of the main comparison groups are presented in table 3. In both meta analyses using multiple studies, the I^2 ^statistic was zero (indicating no heterogeneity beyond that expected by chance) and thus fixed effect models were used.

**Table 3:** Results of meta-analyses

**Intervention**	**Comparison**	**Disorder**	**Follow ** **-up** **period**	**No. ** **Comparisons**	**Total ** **Participants**	**Pooled ** **Effect ** **Size**	**95% CI**
Remote psychotherapy	Control	Depression	0–6 m	7	726	0.44	0.29 to 0.59
Remote psychotherapy	Control	Anxiety-related	0–6 m	3	168	1.15	0.81 to 1.49
Remote psychotherapy	Face-to-face psychotherapy	Depression	0–6 m	1	28	0.55	-0.20 to 1.31
Remote psychotherapy	Face-to-face psychotherapy	Anxiety-related	0–6 m	1	63	-0.11	-0.60 to 0.38
Remote psychotherapy (problem solving therapy)	Remote psychotherapy (stress management)	Depression	0–6 m	1	18	0.38	-0.56 to 1.32
Remote psychotherapy (cognitive behavioral therapy)	Remote psychotherapy (supportive emotion focused therapy)	Depression	0–6 m	1	122	0.39	0.04 to 0.74
Remote psychotherapy (cognitive behavioral therapy)	Remote psychotherapy (supportive emotion focused therapy)	Depression	7 m+	1	117	0.25	-0.12 to 0.62
Remote psychotherapy (exposure therapy)	Remote psychotherapy (relaxation therapy)	Anxiety-related	0–6 m	1	18	1.10	0.10 to 2.10
Remote psychotherapy (exposure therapy)	Remote psychotherapy (relaxation therapy)	Anxiety-related	7 m+	1	14	1.22	0.06 to 2.38

Note: Effect size has been coded so that a positive effect size indicates a study where patients in the intervention group have better outcomes than those in the comparison

**Figure 1:**
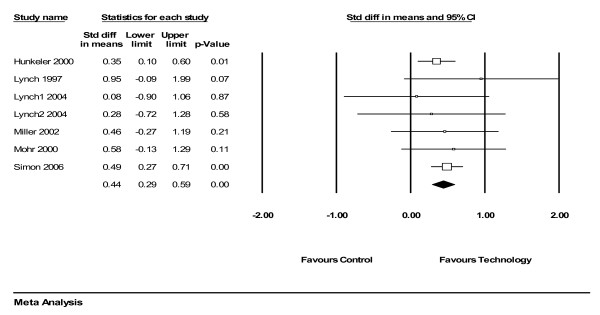
Analysis of technology-mediated therapy versus control (depression).

For depressive disorders, the pooled effect size for remotely delivered therapy compared to control conditions (e.g. waiting list or usual care) was 0.44 (95% CI 0.29 to 0.59), demonstrating a statistically significant 'medium' effect [42] (figure 1).

**Figure 2:**
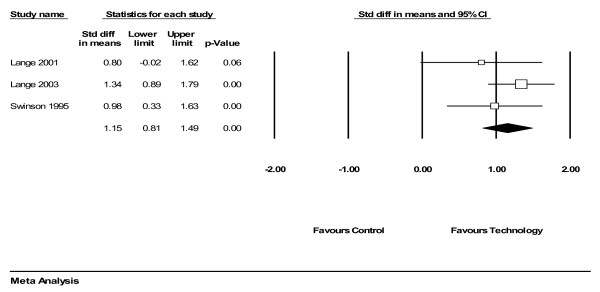
Analysis of technology-mediated therapy versus control (anxiety).

For anxiety-related disorders, remotely delivered therapy demonstrated a 'large' effect compared to control conditions (figure 2, 3 comparisons, n = 168). The pooled effect size was 1.15 (95% CI 0.81 to 1.49) (figure 2).

Comparisons between technology-mediated and face-to-face interventions and comparisons between different forms of technology-mediated therapy were limited by the small number of studies reporting relevant data (table 3).

## Discussion

The aim of this review was todetermine the clinical effectiven ess of psychotherapy delivered by remote communication technologies. Compared to control conditions, technology-mediated therapy demonstrated a 'large' effect for anxiety-related disorders and a 'medium' effect for depression. The majority of included studies suffered at least some methodological limitations and few comparisons of remote versus face-to-face psychotherapy were found.

Overall, the effect size calculations would suggest that remotely delivered psychotherapies do have the potential to be clinically effective, although the magnitude of this effect varies and may be more evident for anxiety than for depression. The comparison of technology-mediated psychotherapy against control conditions for depression produced a pooled effect size of 0.44 in favor of therapy. This finding remains in accordance with an effect size of 0.42 reported by an earlier meta-analysis [60] comparing conventional face-to-face therapy with no treatment controls in depression. A second more recent meta-analysis of face-to-face psychotherapy for depression reports standardized mean differences of 0.63 up to 3 months and 0.56 at 6–9 month follow-up in favor of face-to-face therapy over treatment as usual or waiting list controls [36].

For comparative purposes, meta analyses of lower intensity psychological interventions (e.g. guided self help) for anxiety and depressive disorders report mean effect sizes of between 0.40 and 1.19 [30,31,33,34,36,61], However, these reviews include studies of treatments that, by definition, use technology to reduce rather than mediate professional therapist input. The effects of such interventions have been shown to be greater for some disorders than others [31,33], with certain problems (e.g. anxiety disorders) responding better to self-help packages supported by increased therapist contact [30,33]. The characteristics of patients accessing such treatments are likely to differ substantially from those requiring more traditional and higher intensity therapies, not least in terms of their problem severity and/or motivation for treatment.

A definitive conclusion as to the relative efficacy of technology-mediated versus face-to-face administered psychotherapy can only be drawn from randomized controlled trials comparing the two interventions. A comparison of the efficacy of technology-mediated versus face-to-face psychotherapy was conducted within the present review, with a large but non-significant effect size of 0.55 for depression and a smaller difference of -0.11 for anxiety-related disorders being obtained. However, a shortage of literature limits the utility of this result. Only two of the 13 studies included in the review directly compared the efficacy of face-to-face psychotherapy with an equivalent intervention delivered via more remote means. It should be acknowledged that even in these studies, meaningful comparisons may be difficult. The ability for remote therapy to overcome multiple physical, psychological and geographical constraints means that the clinical populations who may wish to access such treatments may differ markedly from those in face-to-face services.

Ultimately, the published evidence base for clinical effectiveness of psychotherapies delivered solely or largely via remote communication methods is limited, both in size and quality. Therefore, the effect size estimates reported in the current review can at best only be viewed as preliminary. The majority of identified studies were of relatively small sample size with common shortcomings noted in relation to allocation procedures, identification of primary outcome measures and statistical analyses. From a possible maximum score of 42, overall quality ratings for the included studies ranged from 13 to 38 with a median of 22 (SD = 7.33). Despite the above limitations, these scores remain slightly higher than those observed elsewhere, a recent meta-analysis of brief psychological treatments for depression reporting a mean score of 19 (SD 7.3) for 63 controlled trials assessed against the same quality criteria [36]. Given that the financial implications of remote therapy are likely to be a key factor of interest, it is disappointing that no economic data were available.

Attrition rates for the studies ranged from 9 to 73% depending upon the population studied, the nature of the intervention and the length of the follow-up period. This heterogeneity suggests that whilst technology-mediated psychotherapies have the potential to be more effective than delayed or usual care, different treatment modalities may differ in their perceived acceptability. Data from earlier systematic reviews suggest that attrition rates of alternative models of treatment delivery can also vary widely. Randomized trials of face-to-face therapy for depression report post-treatment drop-outs of between 0 and 60% [7]. Trials of written self help materials for anxiety and depressive disorders report comparable figures of between 0 and 61% [62], although attrition from open access online therapy is generally assumed to be higher and may be as much as 99% [63], Once again however, differences in study context, recruitment and sample characteristics can often prevent any direct comparison of attrition rates across different modes of treatment.

Four of the studies included in the review provided a quantitative measure of patient satisfaction, all indicating a preference for, or equivalent satisfaction with, technologically-mediated care as compared to face-to face delivery [56,57] or usual care [51,55]. Two other studies also provided data to suggest that levels of patient satisfaction with remote psychotherapy are high, although in these instances no comparable data from control conditions were available [48,53]. Eighty-three per cent of participants in one trial expressed a favorable attitude towards the use of the telephone to deliver psychotherapy and 75% expressed a desire to continue treatment either now in the future through this medium [53]. However, whether or not these preferences remain consistent across different technologies and communication modalities is unclear. The vast majority of studies included in the present review focused on therapy delivered via the telephone, with very little research effort being directed towards newer and increasingly available resources such as the web. Only one study in the original review examined the efficacy of psychotherapy delivered via videoconferencing and only two studies used the internet, both of which were conducted by the same research team.

A scoping search of literature published since the original searches were undertaken has returned only a small number of additional studies [64-66]. One presents data already included in the present review [64]. Another, examining telephone psychotherapy for depression and conducted as a follow-up to one of trials in the present meta-analysis reports maintenance of clinical benefits when compared to usual care [65]. A third trial that has recently compared face-to-face therapy with a comparable intervention delivered via telepsychiatry reports no significant differences in effect [66].

Whilst the telephone undoubtedly confers specific advantages in terms of its widespread availability and ease of operation, research suggests that videoconferencing may also be particularly suited to psychotherapeutic use. A recent systematic review has shown real time telepsychiatry to represent a highly feasible method of conducting mental health treatments and assessments, often with clinical outcomes and rates of attendance equivalent to those obtained face to face [67]. However, criteria for inclusion and exclusion in this review were not explicitly stated and therefore it is unclear whether or not these observations are based on high quality evidence from randomized trials.

The present review has its limitations. It includes studies examining the effectiveness of psychotherapy delivered via remote communication technology on a scheduled and repeated one-to-one basis and as such, excludes trials examining the effectiveness of more minimal, technology-based interventions. A number of reviews have already been published examining the clinical effectiveness of self-help treatments delivered via technology, and thus the decision was made to conduct a more focused review of optimal relevance to more traditional therapy formats. Nevertheless, publication bias is a problem for any review of controlled trials and given the high dependence on electronic database searching it cannot be certain that unpublished studies do not exist. Due to the small number of studies eligible for the review, funnel plots to assess publication bias were not created in line with recommendations [68]. The analysis treated 'usual care' and waiting list' controls as equivalent to 'no treatment', although it should be noted that previous studies have suggested that effects in usual care may be significantly lower [69]. Translating dichotomous outcomes into standardised effect sizes for the purposes of the meta analysis does allow the most comprehensive summary of the outcome data, but these transformations are based on assumptions which may not always be warranted.

## Conclusion

Technology-mediated psychotherapy provision has the potential to overcome many of the barriers to care associated with more traditional face-to-face interventions. Data suggest that good treatment effects and by implication therapeutic alliance may not be dependent on a patient and therapist being co-located. However, the limited amount of evidence that is available and the restricted number of studies in any one subgroup as yet prevents any definitive conclusions from being drawn. Future research priorities should include overcoming the methodological shortcomings of published work by conducting large-scale trials that incorporate both clinical outcome and more process-orientated measures. In particular, these studies should seek to compare remote psychotherapy with more conventional face-to-face methods by quantifying levels of patient and therapist satisfaction and exploring the potential impact of different modes of communication on therapeutic outcome and examining the quality of the therapeutic alliance that is established,

## Competing interests

The authors declare that they have no competing interests.

## Authors' contributions

PEB participated in the study design, search strategy development, retrieval of articles, screening of articles, quality assessment, data extraction, data synthesis, and writing of manuscript. PB participated in the study design, quality assessment, data extraction, meta-analysis, data synthesis, and writing of manuscript. KL, SG, DR and LG participated in the study design, quality assessment, data extraction, and writing of manuscript. PBarnes participated in the retrieval of articles, screening of articles, quality assessment, data extraction, and writing of manuscript. All authors read and approved the final manuscript.

## Appendix

Electronic search strategy

1. online therapy/or telemedicine/or Diagnosis, Computer-Assisted/or Therapy, Computer-Assisted/

2. telecommunications/or telephone/or videoconferencing/or teleconferencing/

3. internet/or computer assisted instruction/or virtual reality/or Electronic Mail/

4. phone$.mp. or telephone$.mp. or telecommnicat$.mp

5. (text and messag$).mp. or SMS.mp. or (short and messag$).mp.

6. videophone$.mp. or videoconferenc$.mp. or teleconferenc$.mp

7. email.mp. or (electronic and mail).mp. or (electronic and communication).mp.

8.(virtual and reality).mp. or VR.mp.

9.(bulletin and board$).mp. or (discussion and board$).mp. or (discussion and list$).mp.

10. internet.mp. or online.mp. or worldwide web.mp. or web based.mp.

11. Emedicine.mp. or Etherapy.mp. or Ehealth.mp.

12. telemedicin$.mp. or teleconsult$.mp. or telepsychiatry.mp. or telehealth.mp. or teletherap$.mp.

13. (computer and based).mp.or (computer and mediated).mp. or (computer and assisted).mp.

14. (remote and consultation).mp.

15. 1 or 2 or 3 or 4 or 5 or 6 or 7 or 8 or 9 or 10 or 11 or 12 or 13 or 14

16. Psychotherapy/or Brief psychotherapy or Individual Psychotherapy/or Psychotherapeutic Techniques/or Supportive Psychotherapy or or Group Psychotherapy/or Psychotherapeutic Processes/

17. psychotherap$.mp. or psychodynamic therap$.mp. or PDT.mp. or psychoanalytic therap$.mp.

18. Behavior Therapy/or Cognitive Therapy/or Cognitive Behavior Therapy/or Rational Emotive Behavior Therapy/or Behavior Modification/

19. cognitive behavio?r therap$.mp. or cognitive therap$.mp. or behavio?r therap$.mp. or CBT.mp. or cognitive analytic therap$.mp. or behavio?r modification.mp.

20. Relaxation Therapy/or relaxation therap$.mp.

21. Family Therapy/or Interpersonal Psychotherapy/or Psychodynamic Psychotherapy/or Marital Therapy or Group therapy/

22. Interpersonal therap$.mp or IPT.mp.

23. family therap$.mp. or marital therap$.mp. or group therap$.mp. or support group$.mp.

24. GROUP COUNSELING/or PSYCHOTHERAPEUTIC COUNSELING/or EDUCATIONAL COUNSELING/or COUNSELING/or MARRIAGE COUNSELING/or COUNSELING PSYCHOLOGY/or PARENT COUNSELING/or FAMILY COUNSELING/or PATIENT COUNSELING/

25. counsel?ing.mp.

26. Self Help/or Self Management/or exp Self Help Techniques/

27. self treatment.mp. or self help.mp. or self directed.mp. or self management.mp.

28. Behavior Modification/or Relaxation Techniques/or Relaxation Training/

29. relaxation training.mp. or relaxation techniques.mp.

30. psychosocial support.mp. or psychological therap$.mp. or psychological treatment$.mp.

31. exposure therapy.mp.

32. 16 or 17 or 18 or 19 or 20 or 21 or 22 or 23 or 24 or 25 or 26 or 27 or 28 or 29 or 30 or 31

33. 15 and 32

34. limit 33 to English

35. remove duplicates from 34

## Pre-publication history

The pre-publication history for this paper can be accessed here:


